# Waste Powder Biotite as a Factor Enhancing the Flexural Strength of RPC

**DOI:** 10.3390/ma19020276

**Published:** 2026-01-09

**Authors:** Stefania Grzeszczyk, Tomasz Rajczyk, Aneta Matuszek-Chmurowska, Krystian Jurowski, Alina Kaleta-Jurowska

**Affiliations:** 1 Faculty of Civil Engineering and Architecture, Opole University of Technology, Katowicka Street 48, 45-061 Opole, Poland; a.matuszek-chmurowska@po.edu.pl (A.M.-C.); k.jurowski@po.edu.pl (K.J.); a.kaleta-jurowska@po.edu.pl (A.K.-J.); 2 Piława Górna Mine, Kompania Górnicza LLC, Chałubińskiego Street 42, 25-619 Kielce, Poland; tomasz.rajczyk@grupapgs.pl

**Keywords:** waste materials, reactive powder concrete, flexural strength, biotite, microstructure

## Abstract

The advancement of reactive powder concrete (RPC) technology primarily focuses on modifications to its conventional composition. This involves substituting Portland cement (CEM I) with alternative cement types and finely ground mineral additives, as well as replacing quartz aggregate with another type of aggregate. The paper presents an analysis of the properties of RPC obtaining using waste sand and powder generated during the processing of aggregates from migmatite-amphibolite rock. Research into RPC mixtures revealed that in one scenario, replacing quartz powder with waste powder resulted in a significant increase in flexural strength by 23%, although there was a slight decrease in compressive strength by 7%. However, when both quartz powder and quartz sand were substituted with waste powder and waste sand, there was a 14% reduction in compressive strength, while flexural strength increased, albeit to a much lesser extent. The analysis of mineral composition and microstructure of migmatite-amphibolite waste powder and sand revealed that the primary factor contributing to the increase in flexural strength is the presence of biotite in a flake shape form. The microscopy images clearly show hydration products gathering mainly at the rims of biotite flakes and not on their smooth surfaces. The reason could be better availability for hydration products attachment and lower steric hindrance to the rims of single biotite flakes instead of its large packets. Conversely, the reduction in RPC compressive strength, resulting from the substitution of quartz sand with migmatite-amphibolite waste sand, can be attributed mainly to the lower compressive strength of the waste sand itself. Test results indicate that the waste powder generated during the production of migmatite-amphibolite aggregates, which contains fine flakes of biotite, can be utilised as a mineral admixture in concrete, thereby enhancing its flexural strength.

## 1. Introduction

Reactive powder concrete (RPC) is classified as part of the latest generation of ultra-high performance fibre reinforced concrete (UHPFRC), owing to its exceptional mechanical properties and durability. By implementing key modifications in the concrete composition, it is possible to achieve cementitious composites with a compressive strength ranging from 200 to 800 MPa, resulting in enhanced material homogeneity. RPC is typically produced using Portland cement, silica fume, and quartz powder, while the size of the aggregate particles is restricted to a maximum of 600 µm [1,2]. The low water/binder ratio of approximately 0.2, combined with a substantial amount of fine particles, necessitates a greater quantity of advanced superplasticisers [3]. In contrast, the incorporation of steel microfibres significantly improves the flexural and tensile strength of this type of concrete. The RPC technology is based on the principle of achieving optimal particle packing, which ultimately leads to enhanced strength properties and exceptional durability, owing to its minimal porosity [4]. Enhancements in the mechanical properties of RPC can be achieved through the application of heat treatment and pressure during the curing process of the material [5].

Numerous authors have investigated RPC, with some of the earliest studies [1,5] being particularly noteworthy. These studies employed silica fume and ground quartz as reactive powders in RPC mixtures, while quartz sand with a particle size of less than 600 µm was utilised as the aggregate. Given the anticipated high strength of RPC, the cement content is notably significant, ranging from 800 to 1000 kg/m^3^, and should be of a minimum grade of 42.5 [6]. The utilisation of the highest-strength grade 52.5 cement is advantageous [7]. Typically, the proportion of silica fume constitutes 20% of the cement mass. The composition of RPC also includes reactive powders, primarily quartz powder. Additionally, finely ground granulated blast furnace slag, silica fly ash, limestone powder, and glass cullet are incorporated [8]. Kurdowski et al. [7] investigated the impact of various powders, including granulated blast furnace slag, silica fly ash, and corundum powder, which were used to partially replace cement, on the properties of RPC. The RPC incorporating granulated blast furnace slag as a reactive powder achieved the highest compressive strength across different curing conditions. Yazici et al. [9] also demonstrated the beneficial effect of blast furnace slag on strength enhancement in RPC mixes, with slag content of up to 40% of the cement. Additionally, Yazici et al. [10] found that replacing 10% of cement with fly ash resulted in increased strength values for RPC.

Attempts have been made to utilise cements other than CEM I in the production of RPC. These primarily include cements with the addition of granulated blast furnace slag, such as CEM II/B-S and CEM III 42.5 N [11,12,13,14]. Pozzolanic cement CEM IV has also undergone testing [12]. It is noteworthy that the RPC, formulated with blast furnace cement CEM III 42.5 N and cured under standard conditions, achieved a compressive strength of approximately 200 MPa after one year [15].

Research has also been conducted, in which quartz aggregate in RPC mixes was substituted with various other types of aggregates. Richard and Cheyrezy [1], in their conventional RPC formulation, replaced quartz aggregate with steel aggregate of a grain size below 800 µm, enabling them to achieve RPC with a compressive strength of 800 MPa.

In Poland, Zdeb and Śliwiński [16] investigated the feasibility and efficiency of incorporating metallic aggregates into RPC, replacing quartz aggregate either partially (50% by volume) or entirely. They achieved a material with a compressive strength exceeding 300 MPa and a flexural tensile strength of 12.5 MPa. This represents an increase of approximately 25% and 45%, respectively, compared to RPC containing solely quartz aggregate.

Zdeb [17] is the author of a patent that explores the use of unconventional aggregates in RPC, specifically micro-grains of wollastonite with a fibrous morphology, which can replace up to 50% of the quartz aggregate. This innovative approach yields several benefits, including a significant reduction in shrinkage deformation, a twofold increase in flexural strength, and enhanced resistance to brittle fracture.

The authors of the paper [18] substituted the natural fine aggregate in the RPC mix, which was originally composed of ground sand with a particle size ranging from 100 µm to 400 µm, as well as coarse limestone aggregate (0.1–8 mm), with ground clinker aggregate that had a similar particle size distribution to that of natural aggregates. They observed that the incorporation of ground clinker increased the RPC compressive strength by approximately 20 MPa under various curing conditions. The authors attribute this enhancement in strength to the superior strength of the clinker grains and the improved bond strength between the matrix and the clinker aggregate, which reinforces the interfacial transition zone [19].

Serdan Aidyn et al. [20] conducted extensive research on RPC, in which they replaced quartz aggregate with various high-strength aggregates, including corundum, basalt, limestone, sintered bauxite, and granite, all characterised by a similar particle size distribution and a maximum grain size of 4 mm. The authors demonstrated that utilising aggregates larger than those typically used in traditional RPC enables the achievement of RPC with impressive compressive strengths, reaching 200 MPa and beyond under steam curing at elevated temperatures. When autoclaving was employed, the highest compressive strength, exceeding 400 MPa, was attained in RPC containing bauxite aggregate, which exhibited the greatest crushing strength. The authors emphasise that the highest compressive strength was achieved by RPC mixes incorporating aggregates with the highest strength and a rough, porous surface.

Furthermore, it was observed that the flexural strength and fracture energy of RPC were more influenced by the properties of the aggregates than the compressive strength. The highest flexural strength and fracture energy were achieved using corundum aggregate, followed by basalt, bauxite, and granite. In contrast, RPC made with weaker limestone and quartz aggregates, which had smoother grain surfaces, demonstrated the lowest flexural strength and fracture energy. In conclusion, the authors emphasise that attaining satisfactory flexural strength in RPC necessitates the utilisation of high-strength aggregates with a rough surface.

Collepardi and Coppola [21] replaced the fine-ground sand aggregate, which has a maximum grain size of 150–400 µm, in the traditional RPC composition with natural aggregate that has a maximum grain size of 8 mm. A primary objective of their research was to investigate the effect of coarse aggregate on the properties of both fresh and hardened concrete. The authors demonstrated that using aggregate with a larger grain size than that recommended by Richard and Cheyrezy [1] does not reduce the compressive strength of RPC, provided that the water/cement (w/c) ratio in the cement matrix remains unchanged. Consequently, they believe that their test results contradict the proposed model, which attribute the high compressive strength of RPC to the improved homogeneity of the mix in the absence of coarse aggregates. However, it should be noted that the authors of this study do not consider the reduction in flexural strength that occurs when replacing sand with coarser aggregates.

Attempts have also been made to utilise recycled aggregates in the production of RPC. In the study referenced as [22], the authors replaced fine-ground quartz with 25% to 75% recycled aggregate derived from ordinary concrete and RPC, ensuring that the particle size distribution was similar to that of quartz aggregate. An increase in the mechanical properties of RPC was observed with up to 50% recycled aggregate in the mix composition for both types of aggregate. Conversely, the authors of the paper [23] replaced the quartz aggregate in RPC with recycled concrete aggregate, up to 60% by weight, and noted a reduction in the mechanical properties of RPC. Similarly, in the study referenced as [22], when replacing natural aggregate in RPC with recycled concrete aggregate at 75% by weight, a decline in mechanical properties was observed, regardless of whether the waste aggregate was sourced from high-performance concrete (HPC) or ordinary concrete.

The primary objective of the research presented in this paper was to evaluate the potential use of waste sand and powder generated during the production of aggregates from migmatite-amphibolite rock as a replacement for quartz sand and powder in the traditional composition of RPC.

## 2. A General Overview of the Geology of the Piława Górna Mine Deposit

The Piława Górna Mine is situated in the Sudetic Foreland, in the southwestern region of Poland. The mine conducts open-pit extraction and processing of raw materials to produce construction aggregates. The aggregate production process is executed through a process line that includes pre-crushing with a jaw crusher, followed by three consecutive crushing and sorting stages using cone crushers and vibrating screen systems. A vital component of the production process is the dust extraction system, which serves the crushing and sorting equipment areas. Its purpose is to minimise the release of fine particles into the surrounding environment, which are generated during the rock material crushing process. At the final stage of production, fine aggregates undergo a process of fine particle elimination using a cyclone dust collector. Both the dust extraction system for the crushing and sorting equipment and the cyclone dust collector are stages where waste powder is produced, which is subsequently utilised to obtain RPC.

The amount of waste powder generated is approximately 120 tons per day. Due to its dustiness, the stored powder poses an environmental nuisance, making its disposal a priority for the mine. Currently, the waste powder is transported to a dumping site, where it is mixed wet with soil. This process requires significant energy consumption and financial resources.

**Figure 1:**
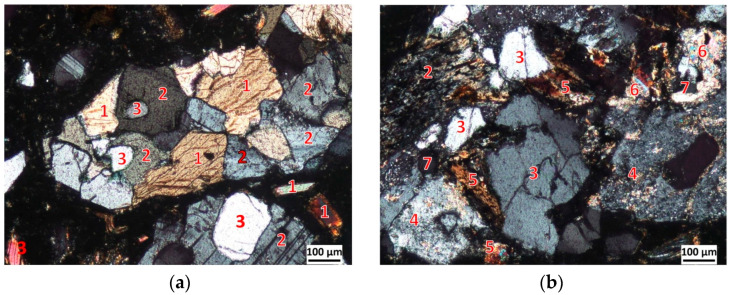
Microscopic image of (**a**) amphibolite; (**b**) migmatite in transmitted polarised light; 1—hornblende, 2—plagioclase, 3—quartz; 4—alkali feldspar, 5—biotite, 6—muscovite, and 7—apatite.

The Piława Górna deposit is predominantly composed of migmatites and amphibolites, which locally contain small veins and pegmatitic nests. The texture of the migmatites features typical varieties, including layered, foliated, ophthalmitic, and dictyonitic forms. Their primary mineral composition consists of quartz, plagioclase, potassium feldspar, and biotite, with subordinate amounts of muscovite, garnet, and accessory minerals such as zircon and apatite. Among the amphibolites, massive varieties are predominant, typically unstructured, with weak foliation being less common, and occasional indistinct lamination or streaking. They exhibit a granoblastic and granolepidoblastic structure, with the main constituents being amphiboles intergrown with plagioclase. In the amphibole-plagioclase paragenesis, biotite is a subordinate component and may undergo significant chloritisation, leading to the formation of epidote and titanium minerals. Garnet can also be present as an accessory component. Locally, within larger masses of amphibolites, hornblende-biotite varieties may be found. Figure 1 shows photographs of amphibolite (a) and migmatite (b) from the Piława Górna deposit.

Table 1 presents the phase composition of the amphibolite rock, and Table 2 presents the phase composition of the migmatite rock, as determined by the Rietveld method. In both migmatites and amphibolites, biotite is a common mineral, typically comprising between 12.5% and 15% by weight. It occurs as elongated, flake-like inclusions within larger grains of the aggregate [24]. Biotite flakes can reach sizes of up to approximately 1.0 mm, with their thickness generally not exceeding one-fifth of the maximum grain diameter. Less frequently, biotite forms local accumulations in the form of clusters. In microscopic images, biotite exhibits a brown colourcolor, which may turn green when chloritised, and displays clear pleochroism. Due to its layered crystal structure, biotite displays exceptional cleavage.

## 3. Materials

### 3.1. Concrete Mix Ingredients

To prepare RPC, CEM I 52.5 R cement (Odra S.A., Opole, Poland) with a specific surface area of 410 m^2^/kg was used. Quartz sand (KiZPPS Osiecznica, Osiecznica, Poland) and waste migmatite-amphibolite sand (Piława Górna Mine, Piława Górna, Poland), with a grain size not exceeding 600 µm, were sourced as aggregates from the aggregate production at the Piława Górna Mine. The finest fractions comprised silica fume (Łaziska Steelworks, Łaziska Górne, Poland), quartz powder (KiZPPS Osiecznica, Osiecznica, Poland), and waste migmatite-amphibolite powder (Piława Górna Mine, Poland). A low water/binder ratio (w/b = 0.2) was achieved by incorporating a polycarboxylate superplasticiser Master Glenium ACE 430 (Master Builders Solutions) at a dosage of 2.5% by cement mass. Steel micro-reinforcement (Baumbach Metall E.S. GmbH, Effelder-Rauenstein, Germany) in the form of steel fibres, measuring 12 mm in length and 0.2 mm in diameter, was included at a volume fraction of 3%.

The particle size distribution of the concrete mix ingredients was assessed using the Mastersizer 3000 laser particle size analyser, covering a range from 0.01 to 3500 µm. The presented results are the average of three tested samples.

**Figure 2:**
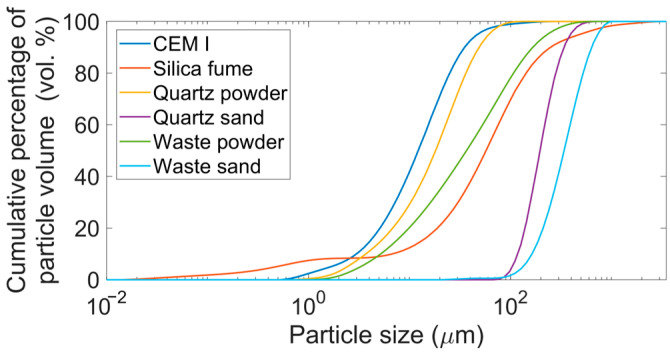
Particle size distribution of the ingredients in the RPC mix.

The composition of the concrete mix was optimised according to the Funk and Dinger curve [25] to enhance its packing density. Figure 2 illustrates the particle size distribution of the ingredients used in the concrete mix. The values Dv(10), Dv(50), and Dv(90) are presented in Table 3.

The waste powder is characterised by a relatively high content of fine fractions. The Dv(50) value is 36 µm, and the Dv(10) value is 5 µm, which means that 50% of the grains have a diameter less than 36 µm, while 10% of the grains have a diameter less than 5 µm.

The waste sand exhibits a noticeably coarser particle size distribution than the quartz sand, as reflected by significantly higher Dv(50) and Dv(90) values (340 µm and 639 µm for waste sand, compared to 203 µm and 341 µm for quartz sand, respectively). Additionally, the waste powder also exhibits a coarser particle size distribution than the quartz powder, with Dv(50) and Dv(90) values of 36 µm and 156 µm, respectively, compared to 18 µm and 48 µm for quartz powder.

### 3.2. Chemical and Mineral Composition of the Ingredients

The chemical composition of ingredients is presented in Table 4. The findings from the mineral composition analysis of the waste sand and powder sourced from the Piława Górna Mine, determined through semi-quantitative calculations using Rietveld refinement, are also summarised in Table 5.

The phase composition of the waste sand and powder (Table 5) indicates that the tested materials predominantly consist of quartz, albite, and biotite. The contents of quartz and albite each exceed 30 wt.%, with albite being significantly more abundant in the powder at 38% compared to 32% in the sand. The biotite content is 15.5% in the sand and 11 wt.% in the powder. Other minerals are present in smaller quantities.

The results of pozzolanic activity tests after 28 and 90 days for the sample of waste migmatite-amphibolite powder, determined in accordance with the EN 450-1 standard [26], showed that the activity index was 59.5% after 28 days and 61.3% after 90 days. This indicates that the waste dust does not meet the standard requirements, which specify values of at least 75% and 85%, respectively. It is worth mentioning that the activity indices for fly ash often exceed 100% [27].

### 3.3. Composition of Concrete Mix

Two RPC mixes were developed using waste materials sourced from the production of construction aggregates at the Piława Górna Mine. These materials included both sand and powder. For comparison purposes, a reference mix was also created, comprising traditional quartz sand and quartz powder (RPC-1). In one variant, quartz sand and quartz powder were fully replaced with waste sand and waste powder (RPC-2), while in another variant, only the quartz powder was substituted with waste powder from the Piława Górna Mine (RPC-3). It is noteworthy that the shape of quartz sand grains differs significantly from that of waste sand grains; quartz sand typically consists of rounded grains, whereas waste sand features sharp-edged forms.

The basic composition of the reference mix (RPC-1) was derived from a previously developed optimal blend using quartz sand and powder, which produced a material with properties detailed in the references [28,29]. In this formulation, the proportions of sand and quartz powder were carefully selected to ensure that the overall aggregate grading closely aligns with the particle size distribution curve proposed by Funk [25], thereby minimising the void content within the aggregate. Table 6 presents the compositions of the RPC mixes.

## 4. Test Methods

The consistency of the RPC mix was evaluated following the method outlined in the EN 1015-3:2000 standard [30], titled “Methods of test for mortar for masonry—Part 3: Determination of consistency of fresh mortar (by flow table).” The consistency was determined by measuring the flow diameter of the RPC mix. The measurement was performed three times.

The concrete compressive and flexural strength was tested in accordance with EN 196-1:2016 standard [31] using 40 mm × 40 mm × 160 mm specimens. The specimens were demoulded after 24 h and cured in water at 20 ± 2 °C before testing. The results presented in the paper are the average of three measurements. The results are presented using mean ± standard deviation.

X-ray diffraction (XRD) analyses were conducted using state-of-the-art PANalytical X’Pert Pro X-ray diffractometer PW3040/60 (PANalytical, Almelo, The Netherlands), equipped with a cobalt anode tube operating at 40 mA and 40 kV, alongside an X’Celerator detector. The analyses covered a broad range of 2Theta values (from 5° to 70°) to ensure the acquisition of a sufficient number of peaks for each phase. Semi-quantitative calculations were performed based on Rietveld refinement, taking into account the preferred orientation of layered silicate crystallites, such as mica and chlorite.

The microstructural analysis of RPC was carried out using a FEI Nova NanoSEM 200 (Thermo Fisher Scientific/FEI, Brno, Cech Republic) field emission scanning electron microscope. X-ray microanalysis (EDS) was performed on selected micro-areas.

RPC porosity tests were conducted using a PoreMaster 60 mercury porosimeter (United States), with pressures ranging from 1 to 400 MPa. The results are presented as differential curves and pore volume distributions for various pore sizes. The results presented in paper are the average of three measurements.

## 5. Test Results and Interpretation

### 5.1. Concrete Mix Consistency

Consistency tests of concrete mixtures using the flow table method showed results for RPC-1, RPC-2, and RPC-3 mixtures of 210 mm, 145 mm, and 210 mm, respectively. The results indicated that the concrete mix, in which quartz powder and quartz sand were replaced with waste powder and sand (migmatite-amphibolite), exhibited the lowest fluidity. In contrast, a higher degree of fluidity was observed in the mix where only quartz powder was substituted with waste powder; in this case, the flow diameter was comparable to that of the reference mix containing quartz sand and quartz powder.

The consistency may be influenced by several factors. The main ones are size, shape, and character of surface of particles, which are the concrete components. Biotite is characterised by a flat, platy (sheet-like) form, which is the primary reason for the reduction in the workability of mortars and concrete mix [32]. Moreover, biotite is a hydrophilic material; the loss of fluidity is attributed to the biotite grain shape and absorption of a large amount of water by the surface in the early stage of cement hydration [33]. The authors [34] also indicate that both workability and slump decrease proportionally with an increase in the biotite content in the aggregate.

### 5.2. Concrete Strength

**Figure 3:**
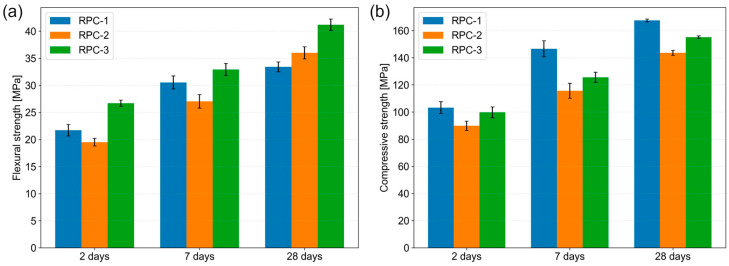
(**a**) Flexural strength of RPC; (**b**) compressive strength of RPC.

The density of hardened RPC was investigated in accordance with the PN-EN 1015-10:2001 standard [35]. The results are as follows: RPC-1 has a density of 2460 kg/m^3^, RPC-2 has a density of 2610 kg/m^3^, and RPC-3 has a density of 2530 kg/m^3^. The results for the compressive and flexural strength of the RPC are presented in Figure 3 and Table 7.

The analysis of RPC strength (Table 7) revealed that the highest compressive strength at the tested intervals of 2, 7, and 28 days was observed in the reference concrete (RPC-1), which contained quartz sand and quartz powder. In contrast, the lowest compressive strength was recorded in the mix where quartz powder and sand were replaced with waste powder and sand. After 28 days, the compressive strength of this concrete was lower by more than 12 MPa (14%).

The higher strength of RPC 1 compared to RPC 2, despite its lower hardened density, can be attributed to differences in the properties of the aggregates and powders used in the two mixes. Waste aggregates and powders applied in RPC 2 differ in density and mechanical properties from the quartz aggregate and quartz powder used in RPC 1. In particular, biotite and hornblende exhibit higher densities than albite due to their chemical composition, while the measured density of waste powders and sands (about 2.9 g/cm^3^) exceeds that of quartz powders and aggregates (about 2.65 g/cm^3^). Moreover, the waste aggregate shows lower hardness and strength than quartz aggregate. These differences could explain the observed strength–density relationship of the investigated concretes.

In contrast, the flexural strength of these concretes exhibits significant variation. A significant increase in flexural strength was observed in the concrete RPC-3, where only quartz powder was substituted with waste powder, compared to the reference sample. After 28 days, the flexural strength increased by approximately 8 MPa (23%), the most notable improvement. For RPC-2, the flexural strength at early stages (2 and 7 days) was slightly lower than that of the reference specimen; however, after 28 days, it surpassed the reference by about 3 MPa (8%). The substantial increase in the flexural strength of RPC specimens containing waste powder can be attributed to the presence of biotite, characterised by its flake-like grains in the fine waste powder. These thin biotite flakes, which differ from the crystal aggregates found in sand, contribute positively to the enhancement of RPC flexural strength (see Section 5.3).

The increase in the flexural strength of RPC was mainly attributed to the elastic biotite flakes. This approach also was based on an analysis of the morphology of minerals present in the waste powder, including hornblende (12.5%) and albite (38.0%). The key differences lie in the crystal structures of these minerals. Hornblende has two cleavage planes intersecting at angles of 60° and 120°, whereas biotite has a single, planar cleavage. Biotite is easily recognisable due to its perfect and uniform cleavage, which allows it to split into thin, elastic flakes, while hornblende does not exfoliate. The elastic properties of biotite flakes have been reported by the authors of [32]. Albite crystals typically exhibit a tabular habit, occurring as granular or massive aggregates, and often show crystal twinning. For this reason, they are not expected to have a significant influence on the elastic properties of the material.

Several studies on the mechanical properties of biotite have been published in the literature [32,33]. These studies indicate a reduction in the compressive strength of mortars and concretes that incorporate biotite-containing aggregates. Additionally, a decrease in the tensile strength of concrete has also been observed. Research into aggregates with biotite has demonstrated a more significant reduction in mechanical properties as the biotite content increases [36]. The authors of paper [24] assert that biotite should be classified as a brittle mineral. According to the findings of paper [37], the elastic modulus of biotite is approximately 20 GPa, which is several times lower than that of quartz. These observations suggest that the presence of biotite in larger structures constitutes a weak link, thereby diminishing the mechanical strength of the resulting cement composites. It is noteworthy that the elastic modulus of the C-S-H phase ranges from 16 to 25 GPa, while that of portlandite is approximately 33 to 94 GPa, depending on the orientation of the crystals [38]. However, it should be mentioned that the studies on biotite’s mechanical properties referenced in papers [32,33] focus on this mineral in the form of large packets found within larger grains of aggregate. In contrast, the increase in the flexural strength of RPC observed by the authors of this paper is attributed to the finely powdered biotite present in the waste powder. In this context, the thin flakes of this material act as a reinforcing factor, enhancing the flexural strength of the RPC.

### 5.3. X-Ray Diffraction and Microstructure of Hardened Concrete

The results of the phase composition analysis of RPC after 2, 7, and 28 days of curing determined using X-ray diffraction (XRD) method; they are presented in Figure 3. A measure of hydration progress can be observed through the amount of crystallised portlandite and the reduced content of unhydrated clinker phases, specifically alite and belite, whose main diffraction lines fall within the 2θ measurement range of 30–35°. The low water/cement ratio (w/c) in RPC results in up to 50% of the cement grains remaining unhydrated. Consequently, the diffraction lines of alite and belite present in the X-ray diffractograms of the specimens, even after 28 days, exhibit significant intensity.

**Figure 4:**
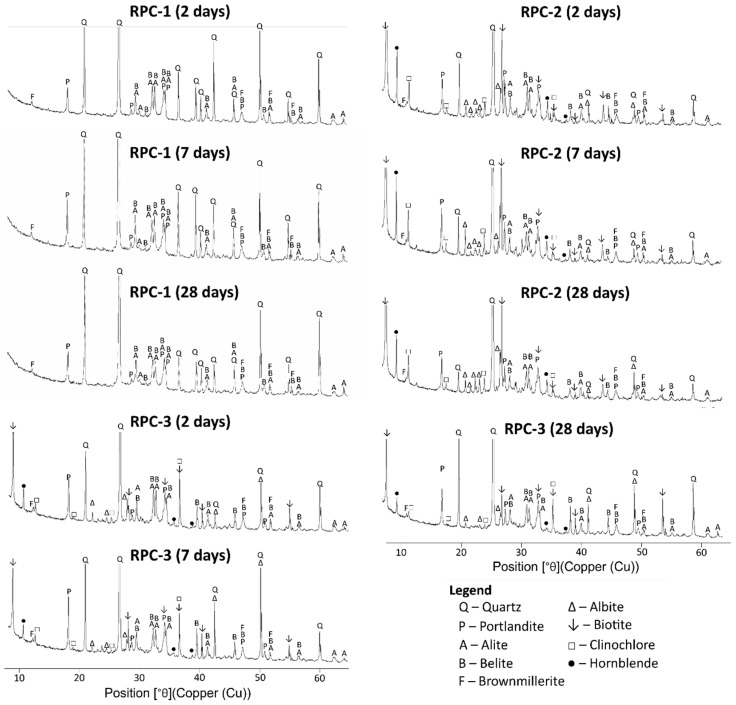
XRD analysis of the RPC-1, RPC-2, and RPC-3 sample after 2, 7, and 28 days of curing.

A comparison of the diffractograms for hardened RPC-1, RPC-2, and RPC-3 after 2, 7, and 28 days (Figure 4) reveals that all RPC specimens contain unhydrated clinker phases, such as alite, belite, and brownmillerite alongside phases formed as a result of cement hydration, notably portlandite (Ca(OH)_2_).

The diffractograms of RPC-2 and RPC-3 display diffraction lines corresponding to the following minerals present in the waste sand and powder from the mine: quartz, biotite, hornblende, clinochlore, and albite. Notably, the intensity of the lines associated with biotite, hornblende, and clinochlore are significantly lower in the diffraction pattern of the RPC-3 samples, with only waste powder, compared to those in the RPC-2 samples, which contain powder and waste sand. This reduction in intensity for biotite, hornblende, and clinochlore is particularly pronounced in the diffractogram of the RPC-3 samples, indicating a marked decrease in the presence of these minerals, relative to the RPC-2 samples containing waste powder and sand.

Over time, the intensity of the diffraction lines corresponding to alite and belite decreases, while the intensity of the diffraction lines associated with portlandite varies. The intensity of the main diffraction line for portlandite (d = 2.628 Å) increases after 7 days and subsequently decreases after 28 days, with the most significant reduction observed in the RPC-1 specimen. In the initial phase, this increase is attributed to the enhanced reactivity of the clinker phases. Conversely, the reduction in intensity of the portlandite line after 28 days, particularly in the RPC-1 specimen, may be due to the reaction of portlandite with the more reactive quartz powder present in this concrete, compared to the waste powder.

**Figure 5:**
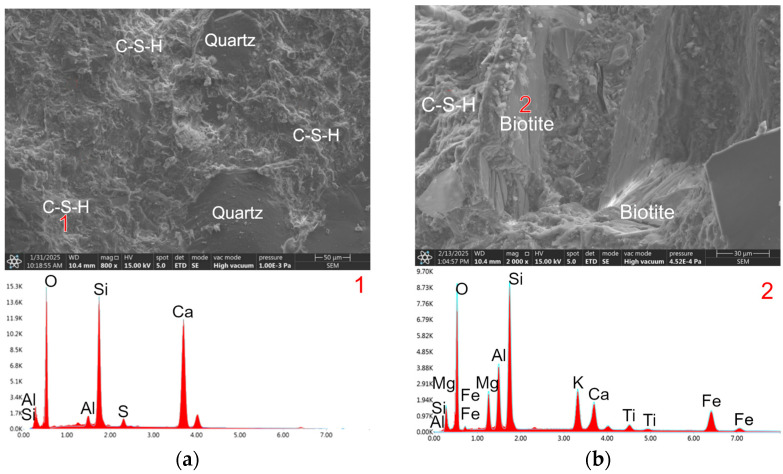
Microstructure of RPC composites: (**a**) RPC-1 after 28 days of curing, showing a compacted C–S–H phase (1); (**b**) RPC-2 after 2 days of curing, showing a flake-like form of biotite (2).

Scanning electron microscope (SEM) observations of the RPC-1 microstructure after 2, 7, and 28 days revealed a significant presence of a dense C-S-H phase, closely adhering to quartz grains within a compact microstructure. The density of this phase increases with hydration time, reaching its optimal state after 28 days (Figure 5a). The high content of the C-S-H phase is primarily attributed to the substantial amount of reactive silica fume incorporated.

**Figure 6:**
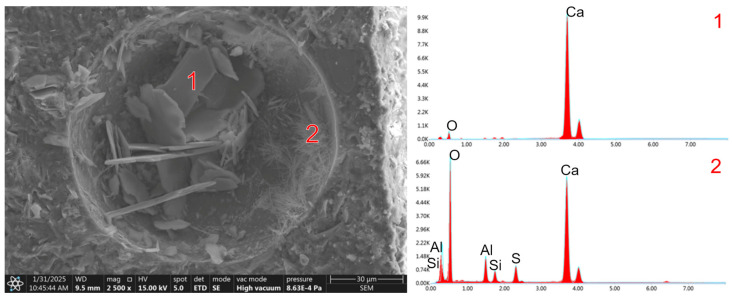
The microstructure of RPC-2 after 28 days of curing reveals prominent large crystals of portlandite (1) and ettringite (2) forming within the pores.

The microstructure of RPC-2, in which quartz powder and sand were replaced with migmatite-amphibolite waste powder and sand, differs fundamentally from that of RPC-1 concrete. It is less compact. Biotite is visible in the form of a packet of flat grains arranged side by side and biotite grains with a smooth surface, on the edges of which cement hydration products in the form of the C-S-H phase are observed (Figure 5b). Numerous pores were observed in the microstructure of this concrete, in which portlandite and ettringite crystals crystallise (Figure 6).

**Figure 7:**
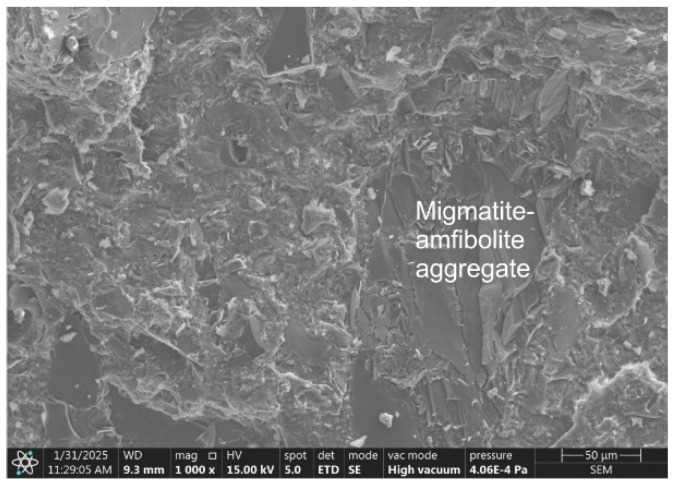
The microstructure o RPC-2 after 28 days of curing. Visible large waste migmatite-amphibolite aggregate grains.

After 28 days of curing of RPC-2 concrete, similarly to the 2-day curing period, the microstructure, in addition to the biotite grain packets, also contained waste migmatite-amphibolite aggregate (Figure 7). As can be seen, the surface of the waste aggregate is smooth, with no cement hydration products observed. The authors of [33] state that the main cause of the reduced compressive, tensile, and flexural strength of concrete with biotite-containing aggregate is primarily the weak bond between the paste and the aggregate. This is due to the limited contact between the cement matrix and the smooth surface of the migmatite-amphibolite aggregate. The authors also attribute the lower strength to the irregular shape of the biotite grains.

**Figure 8:**
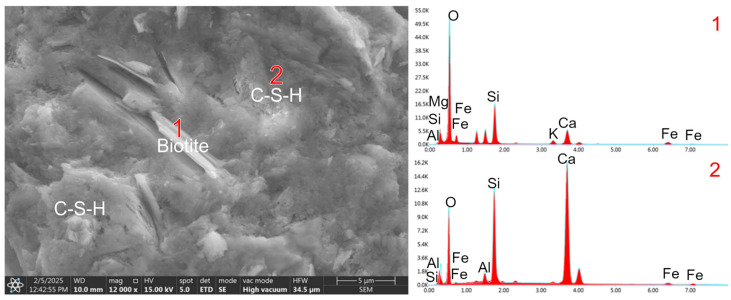
The microstructure o RPC-3 after 28 days of curing reveals visible biotite crystals (1) alongside the adjacent C-S-H phases (2).

**Figure 9:**
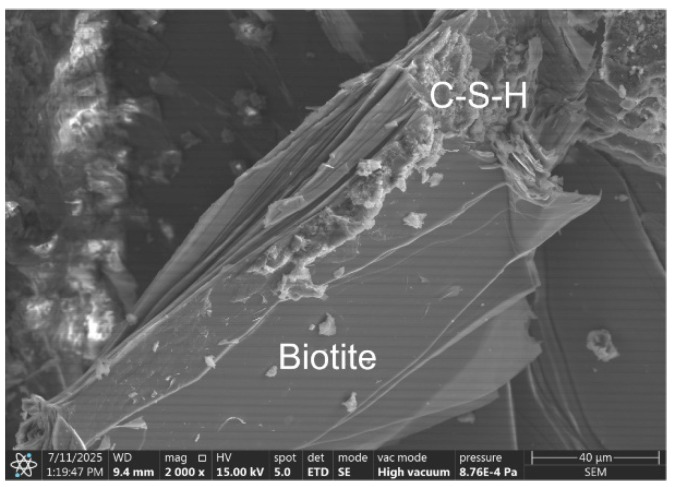
The microstructure o RPC-3 after 28 days of curing. Visible cement hydration products on the edge of packets of biotite flakes.

In the RPC-3 microstructure, where waste migmatite-amphibolite powder was used instead of quartz powder, separated biotite grains with an adjacent C-S-H phase were observed in numerous cases (Figure 8). Packets of plate-like biotite grains with a smooth surface, covered with a negligible number of hydrates, were also observed (Figure 9).

As seen in SEM microscopy images (Figure 5b, Figure 8 and Figure 9), cement hydration products are deposited primarily on the edges of packets and flakes formed by packet separation. They are deposited to a lesser extent on smooth and flat biotite surfaces. Potential defects on the edges of the flakes and flakes facilitate the adhesion of cement hydration products. This phenomenon can be compared to the deposition of cement hydrates on the edges of graphene flakes and the ends of carbon nanotubes, where hydrophilic –COOH, –OH groups are present [39,40]. As a result, this may improve the contact between biotite and the cement matrix. The significant increase in flexural strength using waste powder containing biotite can hypothetically be explained by the greater interaction of the edges of this additive with the cement matrix. Furthermore, separated biotite grains form more flexible flakes during aggregate production than biotite present in the packet.

### 5.4. Porosity

**Figure 10:**
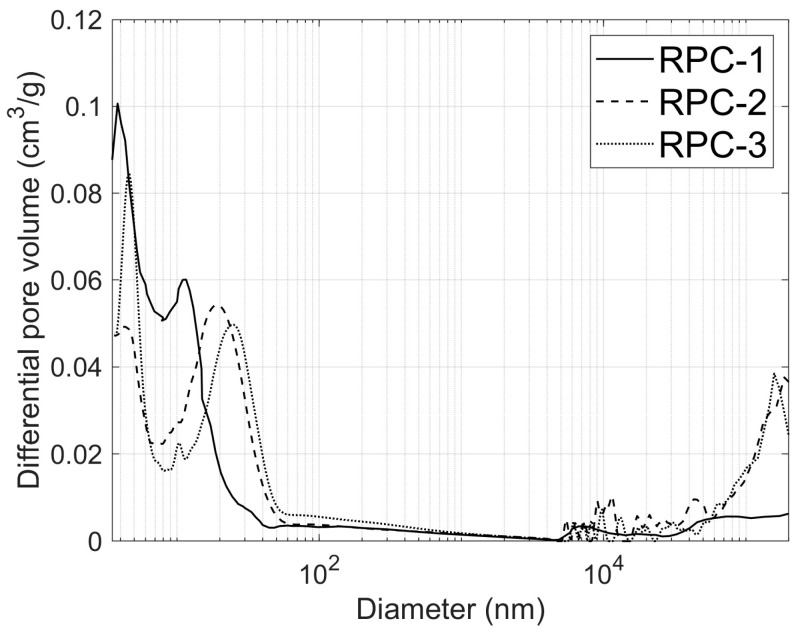
Differential curves of pore volume content as a function of pore diameter after 28 days of curing.

The results of the porosity tests for RPC-1, RPC-2, and RPC-3 concrete are presented in Table 8 and in Figure 10.

As observed in Table 8, the total porosity clearly decreases between 2 and 7 days for all concrete samples. Furthermore, all concretes exhibit a very low pore content in the range of 200–20,000 nm. However, the pore content in the range of 20–200 nm is significantly higher in the concrete containing waste powder and sand (RPC-2 and RPC-3). Additionally, these samples also display a considerably greater number of larger pores (over 20,000 nm), especially RPC-2, compared to the reference concrete (RPC-1). The irregular shape of the migmatite-amphibolite waste sand grains can explain the relatively high content of pores above 20,000 nm in RPC-2 concrete.

## 6. Conclusions

In this work, for the first time, we report on a replacement in RPC powder sand quartz by a waste powder product from a query Piława Górna Mine for the exploration of migmatite-amphibolite rocks, leading to a novel and useful product.

The analysis of the mineral composition of waste sand and waste powder generated during the production of aggregates from migmatite-amphibolite rocks revealed that the primary components of migmatites include quartz, plagioclase, potassium feldspar, and biotite. In amphibolites, amphiboles intergrown with plagioclases are predominant, accompanied by biotite. The biotite content in the waste materials ranges from 12.5% to 15.0% by weight.

The replacement of quartz sand and powder with migmatite-amphibolite waste sand and powder in the RPC mix has a notable impact on the concrete’s strength. What is important, the concrete made with addition of a waste product, including waste powder containing biotite added to cement matrix, showed about 23% higher flexural strength. The biotite content in a waste powder was about 11% by weight.

The increase in flexural strength observed when substituting quartz powder with waste migmatite-amphibolite powder in the RPC mix can be attributed to the presence of fine biotite grains, which exhibit a flake-like morphology. Conversely, the reduction in compressive strength resulting from the replacement of both quartz powder and sand with waste powder and sand is primarily due to the lower compressive strength of migmatite-amphibolite sand compared to quartz sand. It was also noted that the biotite grains in the waste sand formed a more compact structure than those in the powder, where they appeared in a finely pulverised form with a flake-like shape.

The microstructure, obtained by the substitution of quartz powder and sand with waste powder and sand, exhibits significant differences compared to traditional RPC product. The cement hydration products covered rims to a higher degree of fine biotite flakes than their smooth surfaces and, as a result, enforced the cement matrix better.

The results of porosity tests conducted on the cement matrix in RPC corroborated the findings from microscopic examinations. These tests revealed that the RPC containing waste materials, which replaced quartz powder and sand, exhibited higher porosity, mainly resulting from the irregular shape of biotite grains.

## Figures and Tables

**Table 1:** Semi-quantitative analysis of the amphibolite sample calculated using the Rietveld method.

Mineral Name	Chemical Formula	(wt.%)
Ferrian magnesiohornblende	(Na_0.4_K_0.1_) (Ca_1.8_Fe_0.2_) (Mg_3.1_Fe_1.5_Al_0.4_) (AlSi_7_O_22_) (OH)_2_	28.0
Andesine	Na_0.499_Ca_0.491_ (Al_1.488_Si_2.506_O_8_)	34.0
Quartz low	SiO_2_	9.0
Biotite	K_0.946_ Na_0.032_ Fe_1.215_ Mg_1.545_ Mn_0.018_ Ti_0.225_ Al_1.216_ Si_2.784_ O_10_(OH)_2_	15.0
Augite	(Mg_0.73_Fe_0.23_Al_0.02_Ti_0.02_) (Ca_0.86_Na_0.03_Mg_0.02_Fe_0.08_Mn_0.01_) (Si_1.88_Al_0.12_O_6_)	3.0
Orthoclase	KAlSi_3_O_8_	1.0
Clinochlore	(Mg_2.96_Fe_1.55_Fe_0.136_Al_1.275_) (Si_2.622_Al_1.376_O_10_) (OH)_8_	2.5
Ilmenite	Fe_1.04_ Ti_0.96_ O_3_	1.0
Clinozoisite	Ca_2_Al_3_(Si_2_O_7_)(SiO_4_)O(OH)	1.5
Almandine	Fe_1.431_Mg_1.02_Mn_0.039_Ca_0.51_ Al_2_Si_3_O_12_	5.0

**Table 2:** Semi-quantitative analysis of the migmatite sample, calculated using the Rietveld method.

Mineral Name	Chemical Formula	(wt.%)
Oligoclase	Ca_0.277_ Na_0.723_ Al_1.277_ Si_2.723_ O_8_	37.5
Quartz low	SiO_2_	16.0
Biotite	(Na_0.02_ K_0.98_) Fe_1.4_ Mg_0.71_ Ti_0.16_ Al_1.24_ Si_1.36_ O_10_ (OH)_1.64_	12.5
Hornblende I	Na_0.28_ K_0.13_ Fe_1.63_ Mg_3.14_ Al_1.33_ Ti_0.15_ Ca_1.81_ Mn_0.02_ Si_6.92_ O_22_ (OH_1.953_, F_0.047_)	17.5
Hornblende II	Na_0.744_ K_0.039_ Ca_1.84_ Fe_1.08_ Mg_3.33_ Cr_0.08_ Ti_0.12_ Si_6.232_ Al_2.318_ O_22_ (OH)_2_	13.0
Orthoclase	KAlSi_3_O_8_	1.5
Chlorite	(Mg_4.5_ Fe_0.5_) Al_1.84_ Si_3.16_ O_10_ (OH)_8_	2.0

**Table 3:** Dv(10), Dv(50), and Dv(90) values for RPC-1, RPC-2, and RPC-3 components.

Ingredient	Dv(10) (µm)	Dv(50) (µm)	Dv(90) (µm)
Cement	3	12	35
Silica fume	7	56	242
Quartz powder	4	18	48
Quartz sand	121	203	341
Waste powder	5	36	156
Waste sand	164	340	639

**Table 4:** Chemical composition of RPC ingredients (wt.%).

Ingredient	SiO_2_	Al_2_O_3_	Fe_2_O_3_	CaO	MgO	SO_3_	Na_2_O	K_2_O
Cement	21.83	4.38	2.00	65.68	0.93	3.29	0.29	0.32
Silica fume	94.6	0.4	0.5	0.5	0.5	0.8	0.3	1.6
Quartz powder	99.0	0.29	0.05	<0.1	<0.1	-	<0.1	<0.1
Quartz sand	98.6	0.75	0.03	-	-	-	-	-
Waste powder	59.5	16.1	9.33	5.98	3.52	<0.1	2.73	1.34
Waste sand	62.1	15.4	8.99	4.53	4.12	<0.1	2.65	1.29

**Table 5:** Semi-quantitative mineral content of waste sand and powder (wt.%) determined using the Rietveld method.

Mineral Name	Waste Sand	Waste Powder
	(wt.%)	
Quartz low	33.0	30.5
Albite low, calcian	32.0	38.0
Clinochlore	5.0	5.0
Biotite	15.5	11.0
Muscovite	4.5	0.5
Hornblende	2.5	12.5
Orthoclase	3.0	1.5
Almandine	4.0	0.5

**Table 6:** Composition of RPC mix (kg/m^3^).

Ingredient	RPC-1	RPC-2	RPC-3
Cement	901	933	910
Silica fume	180	187	182
Quartz powder	315	0	0
Quartz sand	730	0	737
Waste powder	0	326	319
Waste sand	0	755	0
Superplasticiser	23	23	23
Water	216	224	218

**Table 7:** Compressive and flexural strength of RPC (MPa).

Concrete	Compressive Strength over Time (MPa)		
	2 Days	7 Days	28 Days
RPC-1	103.2 ± 4.37	146.6 ± 5.88	167.4 ± 1.11
RPC-2	89.8 ± 3.40	115.6 ± 5.50	143.5 ± 1.87
RPC-3	99.8 ± 3.99	125.6 ± 3.7	155.2 ± 0.93
	**Flexural Strength over Time** **(MPa)**		
RPC-1	21.7 ± 3.68	30.5 ± 4.53	33.4 ± 3.82
RPC-2	19.5 ± 1.70	27.0 ± 7.64	36.0 ± 6.65
RPC-3	26.7 ± 0.21	32.9 ± 3.46	41.2 ± 1.48

**Table 8:** Porosity of RPC-1, RPC-2, and RPC-3 concrete samples after 2, 7, and 28 days of curing.

Concrete	After Time	Total Porosity	Percentage of Pores (%)				
			<20 nm	20–200 nm	200–2000 nm	2000–20,000 nm	>20,000 nm
RPC-1	2 days	6.1	22.0	57.0	7.0	3.6	10.4
	7 days	4.6	59.9	20.0	4.1	7.7	8.3
	28 days	4.6	77.1	8.0	3.6	2.5	8.2
RPC-2	2 days	6.2	36.0	49.4	3.0	3.2	8.4
	7 days	4.3	25.7	49.5	0.1	4.1	20.5
	28 days	4.4	44.3	22.9	3.4	3.7	25.6
RPC-3	2 days	10.2	26.1	65.2	5.0	0.9	2.7
	7 days	5.6	29.9	45.9	5.4	2.5	16.4
	28 days	6.0	44.3	28.3	4.1	2.7	20.6

## Data Availability

The original contributions presented in this study are included in the article. Further inquiries can be directed to the corresponding author.

## References

[1] Richard P., Cheyrezy M. (1995). Composition of reactive powder concretes. Cem. Concr. Res..

[2] Aïtcin P.C. (2016). Accelerators. Science and Technology of Concrete Admixtures.

[3] Ma J., Orgass M., Dehn F., Schmidt D., Tue N.V. Comparative investigations on ultra-high performance concrete with and without coarse aggregates. Proceedings of the International Symposium on Ultra High Performance Concrete.

[4] Droll K. Influence of additions on ultra high performance concretes-Grain size optimization. Proceedings of the International Symposium on Ultra-High Performance Concrete.

[5] Cheyrezy M., Maret V., Frouin L. (1995). Microstructural analysis of RPC (reactive powder concrete). Cem. Concr. Res..

[6] Zdeb T. (2015). Influence of the physicochemical properties of Portland cement on the strength of reactive powder concrete. Procedia Eng..

[7] Kurdowski W., Garbacik A., Szeląg H. (2009). The influence of reactive powder types on the properties of concrete of reactive powders. Cem. Cem. Lime Concr..

[8] Nehdi M., Mindess S., Aïtcin P.-C. (1998). Rheology of high-performance concrete: Effect of ultrafine particles. Cem. Concr. Res..

[9] Yazici H., Yardimci M.Y., Yigiter H., Aydin S., Türkel S. (2010). Mechanical properties of reactive powder concrete containing high volumes of ground granulated blast furnace slag. Cem. Concr. Compos..

[10] Yazici H., Yigiter H., Karabulut A., Baradan B. (2008). Utilization of fly ash and ground granulated blast furnace slag as an alternative silica source in reactive powder concrete. Fuel.

[11] Srinivas A., Craig M.N. Ultra-High Strength Concrete Mixtures Using Local Materials. Proceedings of the 2010 Concrete Sustainability Conference.

[12] Chang T.P., Chen B.T., Wang J.J., Wu C.S. (2008). Performance of Reactive Powder Concrete (RPC) with different curing conditions and its retrofitting effects on concrete member. Concrete Repair, Rehabilitation and Retrofitting II.

[13] Cwirzen A., Penttala V., Vornanen C. (2008). Reactive powder based concretes: Mechanical properties, durability and hybrid use with OPC. Cem. Concr. Res..

[14] Herold G., Müller H.S. Measurement of porosity of ultra-high strength fibre reinforced concrete. Proceedings of the International Symposium on Ultra High Performance Concrete.

[15] Grzeszczyk S., Janus G. (2020). Reactive powder concrete with lightweight aggregates. Constr. Build. Mater..

[16] Zdeb T., Śliwiński J. (2012). Beton z proszków reaktywnych z kruszywem metalicznym. Monografie Technologi Betonu.

[17] Zdeb T. (2021). Application of Wollastonite as a Mineral Additive for Concrete Matrices from Reactive Powder Concrete (RPC).

[18] Monosi S., Pignoloni G., Collepardi S., Troli R., Collepardi M. (2000). Modified Reactive Powder Concrete with Artificial Aggregates. SP-195 Sixth Canmet/ACI Conference on Superplasticizers and Other Chemical Admixtures in Concrete.

[19] Aitcin P.C. (2003). The art and science of durable high-performance concrete. Ind. Ital. Del Cem..

[20] Aydin S., Yazici H., Yardimci M.Y., Yiğiter H. (2010). Effect of Aggregate Type on Mechanical Properties of Reactive Powder Concrete. ACI Mater. J..

[21] Collepardi S., Coppola L., Troli R., Collepardi M. (1997). Mechanical properties of modified reactive powder concrete. ACI Spec. Publ..

[22] Salahuddin H., Qureshi L.A., Nawaz A., Raza S.S. (2020). Effect of recycled fine aggregates on performance of Reactive Powder Concrete. Constr. Build. Mater..

[23] Mohammed S., Karim A.A., Thamer A.Z. (2023). Experimental study on the effect of size, type, and replacement ratio of recycled aggregate on the mechanical properties of reactive powder concrete. Misan J. Eng. Sci..

[24] Tagnit-Hamou A., Saric-Coric M., Rivard P. (2005). Internal deterioration of concrete by the oxidation of pyrrhotitic aggregates. Cem. Concr. Res..

[25] Funk J.E., Dinger D.R. (2013). Predictive Process Control of Crowded Particulate Suspensions: Applied to Ceramic Manufacturing.

[26] (2012). Fly Ash for Concrete—Part 1: Definition, Specifications and Conformity Criteria.

[27] Baran T. (2019). The activity indexes of siliceous flyashes tested with standard Portland cements CEM I 42.5R, with different phase composition. Cem. Wapno Beton.

[28] Zdeb T., Śliwiński J. (2010). Beton z proszków reaktywnych-właściwości mechaniczne i mikrostruktura. Bud. Technol. Archit..

[29] Zdeb T. (2009). Wpływ Składu i Technologii Wykonania na Wybrane Właściwości Betonów z Proszków Reaktywnych.

[30] (2007). Methods of Test for Mortar for Masonry—Part 3: Determination of Consistence of Fresh Mortar (by Flow Table).

[31] (2016). Methods of Testing Cement—Part 1: Determination of Strength.

[32] Wakizaka Y., Ichikawa K., Nakamura Y., Anan S. (2001). Deterioration of concrete due to specific minerals. Environ. Econ..

[33] Xue H.-L., Han C.-Q., Chen M.-L., Fan G., Zhou J.-W. (2022). Improving mechanical properties of manufactured sand concrete with high biotite content: Application of magnetic separation process and equipment optimization. Constr. Build. Mater..

[34] Mueller O.H. (1971). Some aspects of the effect of micaceous sand on concrete. Civ. Eng. S. Afr..

[35] (2007). Methods of Test for Mortar for Masonry—Part 10: Determination of Dry Bulk Density of Hardened Mortar.

[36] Cacciari P.P., Futai M.M. (2019). Effects of mica content on rock foliation strength. Int. J. Rock Mech. Min. Sci..

[37] Espinoza W.F., Pereira J.-M., Kneafsey T., Dai S. (2023). Mechanical and creep properties of granitic minerals of albite, biotite, and quartz at elevated temperature. Geomech. Energy Environ..

[38] Manzano H., Dolado J., Ayuela A. (2009). Elastic properties of the main species present in Portland cement pastes. Acta Mater..

[39] Chełmecka E., Pasterny K., Kupka T., Stobiński L. (2012). OH-functionalized open-ended armchair single-wall carbon nanotubes (SWCNT) studied by density functional theory. J. Mol. Model..

[40] Chełmecka E., Pasterny K., Kupka T., Stobiński L. (2012). DFT studies of COOH tip-functionalized zigzag and armchair single wall carbon nanotubes. J. Mol. Model..

